# A decade of travel-associated malaria at King Fahad Hospital of the University in the Eastern Province of Saudi Arabia

**DOI:** 10.1038/s41598-022-04996-4

**Published:** 2022-01-19

**Authors:** Ashwaq Alanazi, Bayan Ahmad Hasan Almusailhi, Gheed K. Bamousa, Nabaa H. Alhawashim, Nourah M. Alotaibi, Sumiyah AlShamekh, Basavaraja Channabasappa Hunasemarada, Reem Y. Al Jindan, Ayman A. El-Badry

**Affiliations:** 1 College of Medicine, Imam Abdulrahman Bin Faisal University, Dammam, Saudi Arabia; 2 Department of Microbiology, College of Medicine, Imam Abdulrahman Bin Faisal University, Dammam, Saudi Arabia

**Keywords:** Microbiology, Diseases

## Abstract

Travel-associated malaria is a health hazard, even in non-malaria endemic regions. This is a hospital-based retrospective study of 12,931 febrile patients who presented at King Fahad Hospital of the University (KFHU) from January 2009 to December 2019. Patients either returning from malaria endemic countries and/or for whom malaria was suspected, had blood films microscopically screened for malaria parasites. Malaria prevalence was very low in febrile patients attending KFHU. Out of the 12,931 febrile patients, 0.63% (n = 81) were malaria positive, all travel-related, except for one case of transfusion malaria. Indian nationals were the most infected (29.6%, n = 24), followed by Sudanese nationals (24.7%, n = 20). *P. falciparum* (47%, n = 38) and *P. vivax* (42%, n = 24) were the predominant species. The majority of *P. falciparum* (64.5%, n = 20) cases were from African nationals and the majority of *P. vivax* (72.7%, n = 24) cases were from Asia. The highest percentage of malaria patients were adult (90%, n = 73), males (85.2%, n = 69), ages ranged from 6 to 65, with a mean of 34.6 years. Most of the malaria cases presented at the emergency room (ER), only 3 required critical care. Only sex, hospitalized in-patient (IP) and attendance at ER were statistically associated with malaria. In the presence of a potential vector, travel-associated malaria in non-malaria endemic areas should be monitored to guide control strategies.

Author summary: Malaria is a neglected potentially fatal tropical mosquito-born disease. Travel-associated malaria is a health hazard, even in non-malaria endemic regions. In spite of previous efforts to estimate malaria prevalence, morbidity and mortality in Saudi Arabia in the last decade, there have been no studies that determine the prevalence of malaria in Al-Khobar, Eastern Province of Saudi Arabia. Malaria prevalence was very low in febrile patients (81/12,931) attending King Fahad Hospital of the University over a decade. Cases were all travel-related, except for one case of transfusion malaria. Indian nationals were the most infected (29.6%), followed by Sudanese nationals (24.7%). *P. falciparum* (47%) and *P. vivax* (42%) were the predominant species. The majority of *P. falciparum* (64.5%) cases were from Africa and the majority of *P. vivax* (72.7%) cases were from Asia. No patient factors predicted malaria in febrile travelers. In non-malaria endemic areas, in the presence of a potential vector, patients with acute fever coming from endemic areas or having received blood transfusion, should be screened for travel-associated malaria to guide control strategies.

## Introduction

Malaria, a lethal vector-borne protozoan disease, is transmitted by the female anopheles mosquito. It is a prevalent disease in subtropical and tropical countries, and an increased health hazard for travelers. It is a resurgent disease even in non-malaria endemic areas, such as the Eastern Province of Saudi Arabia^1^.

Malaria is one of the major infections and causes of hospitalization for febrile travelers returning from subtropics and tropics^2–4^. Acute fever or acute febrile illness is a rapid onset of undifferentiated fever of 38 °C or higher for 2–7 days and can persist up to 3 weeks without localizing organ-specific symptoms or signs caused by many pathogens, malaria being the major cause in tropics^5^.

More than 3 billion people globally are at risk of contracting malaria. In 2019, the world health organization (WHO) reported that infected malaria cases reached 229 million cases in 2019 as compared to 218 million cases in 2015 worldwide, with 409 thousand recorded deaths in 2019. In the Eastern Mediterranean region, the recorded cases in 2019 were 5 million^6^. Saudi Arabia is considered to belong to Eastern Mediterranean region by WHO^6^.

In the last decade there has been a worldwide reduction in many endemic countries, which suggests that the efforts to control the disease are working, and if sustained, malaria may be eliminated, specially by *P. falciparum*. Due to aspects of parasitic biology, the elimination of *P. vivax* malaria presents more challenges. Though the reduction in morbidity and mortality rates of malaria has slowed since 2016^6^, there is an increase in the rate of imported malaria in non-endemic and malaria free areas due to many socio-demographic factors, including an increase in population movement mainly due to travel, working expatriates, and migration^6,7^.

There are five *Plasmodium* (P.) parasite species known to cause human malaria, two of which pose a serious threat to humans. *P. falciparum* and *P. vivax* are the two species known to be widespread and cause severe disease in infected patients. *P. falciparum* accounts for most of the cases reported by WHO in Africa, South-East Asia, Western Pacific and Eastern Mediterranean regions. However, *P. vivax* is the predominant species in the Americas (76%), and the second most predominant species in South-East Asia (46%). In the Eastern Mediterranean region, both *P. falciparum* and *P. vivax* parasites are prevalent; about three quarters of the cases are attributed to *P. falciparum*^6^.

Many countries around the world face the challenge of identifying travel-related malaria, Saudi Arabia being one of these countries. Imported cases from endemic countries are also a threat to any malaria elimination effort by Saudi Arabia health authorities as result of the presence of a large number of expatriates coming from malaria endemic areas^8^. Added to this, many Saudi nationals travel to malaria endemic countries.

Because of the increase in international and national mobility toward malaria endemic regions, there is an increased risk of re-introduction of malaria in areas such as the Eastern Province of Saudi Arabia (where malaria has not been endemic since 1978 as a result of ongoing malaria control program^9^) due to the presence of *Anopheles* spp. mosquitos, a potential malaria vector. Many *Anopheles* spp. mosquitos were recorded in Eastern Province of Saudi Arabia. These species include *Anopheles stephensi* and *Anopheles gambiae*, the primary malaria vectors in Saudi Arabia, in addition to *Anopheles fluviatilis*, *Anopheles multicolor* and *Anopheles arabiensis* which have not been incriminated for Malaria transmission in Saudi Arabia^10,11^.

In spite of previous efforts to estimate malaria prevalence, morbidity and mortality in Saudi Arabia, there have been no studies in the last decade that demonstrate the prevalence of malaria in King Fahad Hospital of the University (KFHU), Al-Khobar, at the center of Eastern Province of Saudi Arabia and serving an estimated population of 1.25 million.

## Materials and methods

A hospital-based retrospective study was done, including all febrile patients who presented at KFHU from January 2009 to December 2019 for travelers returning from malaria endemic areas or for whom malaria was clinically suspected; their Giemsa stained thin and thick blood films were screened for detection and speciation of malaria parasites microscopically by malaria experts, parasite density was determined according to WHO recommendations^12^. Quality control of blood films was carried out. Patient was considered febrile if his body temperature was 38 °C or higher. Using a standard form, the collection of patient’s characteristics was done from a retrospective review of the medical records, the lab reports and discharge notes of all patients, after obtaining ethical approval and KFHU permission. Related patient data, including date of diagnosis, test results, patient residence, sex, age, nationality, visited clinic, history of travel, history of blood transfusion, associated symptoms, comorbidity and treatment regimen, were recorded for each patient.

Data was entered, coded, tabulated and malaria prevalence as well as the association of patient characteristics with occurrence of malaria were analyzed using IMB SPSS software version 26. The qualitative and quantitative data were presented, to compare means, the Fisher exact test and the Chi-square test were used as appropriate. *P*-values < 0.05 provide evidence for a statistically significant difference.

The association between patient factors and occurrence of malaria were assessed. To identify malaria predictors, all patient variables showing significant association with malaria infection were entered into logistic regression models using ENTER method and prediction was measured by odds ratio and considered significant if *P*-value < 0.05. ENTER method is a simultaneous standard method of entry of all independent variables into the regression equation at the same time^13^. We used pre-existing recorded observational patient data at the information technology department in KFHU that was obtained from a non-random sample of the population^14^, thus the results of the used statistical tests are simply indicative.

Ethical approval of the current study was received from the Institutional Review Board (IRB #: UGS-2019-01-334), deanship of scientific research and postgraduate studies of Imam Abdulrahman Bin Faisal University. A permission letter was provided by the microbiology laboratory and microbiology department to use patient data obtained from the information technology (IT) department in KFHU. The confidentially of all patients was preserved since no details identifying the identity of the patients were used.

### Ethical approval

Ethical approval of the current study was received from the Institutional Review Board (IRB #: UGS-2019-01-334), deanship of scientific research and postgraduate studies of Imam Abdulrahman Bin Faisal University. All methods were carried out in accordance with relevant guidelines and regulations. Informed consent was waived by institutional ethics committee because this is retrospective study.

## Results

Out of 12,931 febrile patients clinically suspected for malaria, 81 patients were malaria positive by microscopic examination of blood smear, with 0.63% prevalence rate. The body temperature of all malaria patients of the study was 38 °C and higher, the range of body temperature was between 38 and 40.1 °C and the average was 39.4 °C.

The descriptive analysis of related patient data were presented in Table 1.

**Table 1:** Descriptive statistics of febrile patient data and their association with malaria infection.

			Malaria			*P* value*
			Positive, N	Negative, N	Total, N (%)	
Sex	Male		69	6320	6389 (49.4)	0.0001
	Female		12	6530	6542 (50.6)	
Age group*	Infant (< 1 year)		0	4	4 (0.03)	0.276
	Child (1– < 12)		4	1326	1330 (10.3)	
	Adolescent (12– < 20)		4	1249	1253 (9.7)	
	Young adult (20– < 40)		47	5640	5687 (44.0)	
	Middle age adult (40– < 60)		23	4020	4043 (31.27)	
	Old adult (60– < 70)		3	469	472 (3.6)	
	Elderly (> 70)		0	142	142 (1.1)	
Hospital presentation**	ER		47	5844	5891 (45.5)	0.0001
	CC		3	5461	5464 (42.3)	
	IP		30	456	486 (3.7)	
	OP		1	1089	1090 (8.5)	
Nationality	Saudi		9	1609	1618 (12.5)	0.702
	Non-Saudi	India	24	1727	1751 (13.5)	
		Sudan	20	750	770 (5.9)	
		Pakistan	11	632	643 (5.0)	
		Nigeria	7	76	82 (0.6)	
		Kenya	2	63	65 (0.5)	
		Nepal	2	9	11 (0.1)	
		Egypt	1	2628	2629 (20.3)	
		Ethiopia	1	126	127 (1.0)	
		Ghana	1	14	15 (0.1)	
		Other***	3	5218	5221 (40.4)	
		Total	72	11,241	11,313 (87.5)	
Total			81 (0.63%)	12,850 (99.37%)	12,931 (100.0%)	

Proportions of categorical variables were compared using the Chi-square test.

(*) *P-*value < 0.05 is significant (comparing between malaria positive). (**) ER (emergency room), CC (critical care), IP (hospitalized in-patient), OP (out-patient). (***) other are non-Saudi and their nationalities were not recorded.

The mean age of malaria infected patients was 34.6 years, and ranged from 6 to 65 years; 85.2% (69/81) of them were males and 14.8% (12/81) were females, the male/female ratio was 5.75:1 (Table 1). Malaria was common among young adults (58%, 47/81), followed by middle age adults (28.4%, 23/81). Children (4.9%, 4/81), adolescents (4.9%, 4/81), and the elderly (3.7%, 3/81) were the least affected age groups (Table 1).

A total of 12,850 (99.37%) patients were malaria negative, their age ranged from 1 to 105 years, with a mean of 34.2 years; 49.2% (6530/12,850) of them were males and 50.8% (6320/12,850) were females, the male/female ratio of 1:1 (Table 1). The distribution of age groups of malaria negative febrile patients was shown in Table 1, most of them were young adults (43.9%, 5640/12,850).

The highest frequency among malaria positive blood samples was from the emergency room (ER) (58%, 47/81), followed by blood samples from hospitalized in-patients (IP) (37%, 30/81) (Table 1).

**Figure 1:**
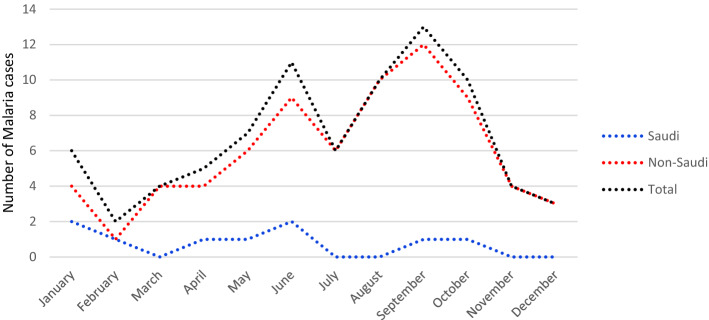
Monthly distribution of malaria positive cases.

**Figure 2:**
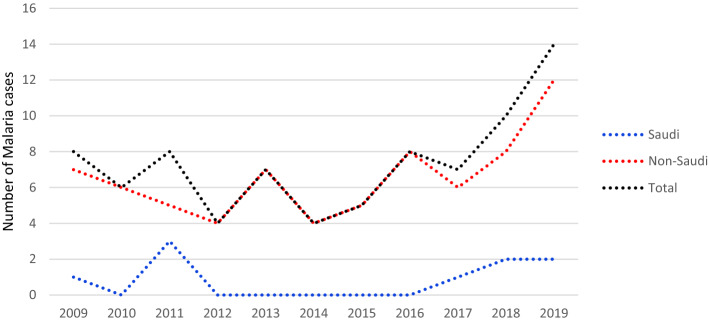
Annual distribution of malaria positive cases.

Malaria cases were recorded throughout the year with a peak in September (Table 2). There was annual and monthly seasonality (Table 2), with an increase in recording of cases late summer and early fall. Most of the cases were recorded in 2018 and 2019. After 5 years of zero malaria cases among Saudi population, there has been a small surge of 5 cases (55.6%) over the last 3 years, without history of travel outside of the country (Figs. 1 and 2).

**Table 2:** Seasonality of febrile patients and its association with malaria infection.

	Malaria			*P* value*
	Positive	Negative	Total, N (%)	
**Seasonality**				
January	6	928	934 (7.2)	0.262
February	2	818	820 (6.3)	
March	4	782	786 (6.1)	
April	5	1016	1021 (7.9)	
May	7	1128	1135 (8.8)	
June	13	1112	1125 (8.7)	
July	5	814	819 (6.3)	
August	10	1042	1052 (8.1)	
September	14	1731	1745 (1.5)	
October	9	1549	1558 (12.0)	
November	4	1034	1038 (8.0)	
December	2	896	898 (6.9)	
Total	81 (0.63)	12,850 (99.37%)	12,931 (100.0%)	

Proportions of categorical variables were compared using the Chi-square test.

* *P-*value < 0.05 is significant.

Except for one case of transfusion malaria, all study cases were travel related malaria. Non-Saudi individuals were returning from their respective malaria endemic countries of origin. Three *Plasmodium* species were detected in malaria infected patients including *P. falciparum* species, *P. vivax* species and *P. ovale* species, with *P. falciparum* species the most frequent species (38 cases), followed by *P. vivax* species (34 cases) (Table 3).

**Table 3:** Patient nationality distribution according to *Plasmodium* species and parasitaemia%.

Nationality		Plasmodium species*				Parasitaemia%			Total N (%)
		*P.f*	*P.v*	*P.f* + *P.v*	*P.v* + *P.o*	< 1	1–5	> 5	
**Saudi**		7	1	1	0	9	0	0	9 (11.1)
**Non-Saudi**	India	7	14	3	0	21	2	1	24 (29.6)
	Sudan	13	7	0	0	17	3	0	20 (24.7)
	Pakistan	1	9	1	0	11	0	0	11 (13.6)
	Nigeria	4	2	1	0	5	2	0	7 (8.9)
	Kenya	1	0	0	1	2	0	0	2 (2.5)
	Nepal	0	1	1	0	1	1	0	2 (2.5)
	Egypt	1	0	0	0	1	0	0	1 (1.2)
	Ethiopia	1	0	0	0	0	1	0	1 (1.2)
	Ghana	1	0	0	0	1	0	0	1 (1.2)
	Other**	2	0	1	0	2	1	0	3 (3.7)
	Total	31	33	7	1	61	10	1	72 (88.9)
Total		38	34	8	1	70	10	1	81 (100%)

(*) *P.f* (*P. falciparum*), *P.v* (*P. vivax*), *P.o* (*P. ovale*). (**) other are non-Saudi and their nationalities were not recorded.

Out of the malaria positive patients, 9 were Saudi and 72 were non-Saudi. Among the 9 Saudis infected by malaria, one case was transfusion malaria, and 8 cases were travel-related, 7 had travelled to the malaria endemic area in southwestern Saudi Arabia, and only one was returning from international travel. There were 8 males, 7 of them infected by *P. falciparum* and one had *P. vivax,* and one pregnant woman had mixed *P. falciparum* and *P. vivax* transfusion malaria, her pregnancy resulted in an abortion, the blood donor was not identified in the patient’s records (Tables 1 and 3).

Within patient data, only sex and hospital presentation (hospitalization (IP) and attendance at ER) were statistically associated with malaria and were subjected for univariate analysis using logistic regression. Being a hospitalized patient (IP) or a patient attending ER were predictors for the probability of having malaria, (OR = 0.6, 8.8 and 71.6 respectively) (Tables 1 and 4).

**Table 4:** Sex and hospital presentation of febrile patients associated with malaria infection.

		Frequency			OR	95% CI	*P* value*
		Positive	Negative	%^#^			
Sex	Male/Female	69/12	6320/6530	1.09/0.18	0.604	0.31–1.19	0.140
Hospital presentation	ER/OP	47/1	5844/1089	0.81/0.09	8.758	1.21–63.55	0.032
	CC/OP	3/1	5461/1089	0.05/0.09	0.598	0.62–5.76	0.657
	IP/OP	30/1	456/1089	6.45/0.09	71.645	9.74–526.94	0.0001

ENTER method of logistic regression model was used. Data presented as n, (^#^) % of malaria positive within the same group, (*) *P-*value < 0.05 is significant.

From the 81 malaria cases, around 40% (32/81) of the patients required hospitalization, among them 3 cases were *P. falciparum* and required critical care. All malaria patients were treated with the standard malaria treatment according to the guidelines of malaria treatment policy in Saudi Arabia^13^, with 100% cure rate.

## Discussion

Despite previous efforts to estimate malaria prevalence, morbidity and mortality in Saudi Arabia over the last decade, there have been no studies that specifically demonstrate the prevalence of malaria in Al-Khobar, Eastern Province of Saudi Arabia. In our hospital-based study of 12,931 febrile patients who attended KFHU, there was a very low malaria prevalence of 0.63%. Most of the confirmed malaria cases were imported malaria from endemic countries; however, in spite of a large reduction of indigenous cases in 2019 as compared to 2017^6^, there remains a very low number of indigenous malaria cases in Saudi Arabia. Our findings indicate that malaria screenings should include indigenous cases and not be restricted to travelers and expatriates from malaria endemic areas.

The large number of foreign patients coming to Saudi Arabia from endemic areas can be explained by the large number of expatriates residing in the Eastern Province of Saudi Arabia who are from endemic malaria areas^15^.

Though Saudi Arabia witnessed a drastic decline in the prevalence of malaria, with zero deaths in the last decade due to effective nationwide elimination program^6,16^, imported malaria may potentialize indigenous malaria transmission, particularly in the presence of the anopheles mosquito vector.

Malaria transmission is ongoing in over 87 tropical and subtropical countries, of which more than 90% of the cases are in Africa. WHO estimated 229 million malaria cases 2019, with a majority of cases from India and Sub-Saharan countries^6^. These high numbers of malaria patients from Indian sub-continents and Sub-Saharan Africa, returning to Saudi Arabia after visiting their countries, are probably the reason why in our study most of the positive cases in KFHU were from the Indian sub-continents and Sub-Saharan Africa, especially India (29.6%), Sudan (24.7%) and Pakistan (13.6%) respectively. Except for one case of transfusion malaria, all were travel related malaria. It has been described in previous Saudi Arabian studies that the vast majority of cases are imported^16,18,19^. Yemen has been identified as one of the large sources of imported malaria in Saudi Arabia^20^, while, none of the imported malaria cases in our study were from Yemen, most our indigenous cases had a history of travelling to the southern border, which is shared with Yemen, where malaria is hyperendemic and prevalent^16^.

In our study, *P. falciparum* (38/81) was the predominant malaria species, both in Saudi and non-Saudi patients, followed by *P. vivax* (34/81) and 9 cases which had mixed infection. Our findings are consistent with previous reports for different regions from Saudi Arabia^21,22^. The majority of *P. falciparum* (64.5%) cases were from Africa and majority of *P. vivax* (72.7%) cases were from Asia. This predominance agrees with worldwide malaria facts, which can be explained by the country of origin of expatriates and endemicity of *Plasmodium* species^6^. According to 2020 WHO World Malaria Report, *P. falciparum* is most prevalent species in many parts of the world, including Africa, Eastern Mediterranean region and South-East Asia, accounting for 99.9%, 73% and 53% of detected malaria species respectively. *P. vivax* is second most predominant malaria species worldwide, with about half of global *P. vivax* cases from South-East Asia, mainly India and Pakistan^6,23^.

Although the WHO worldwide fact sheet in 2020 stated that majority of affected patients were children < five years, making them the most vulnerable group affected by malaria, in our study, young adults were the most affected age group, followed by middle age, with mean age of 34.59 ± 11.947 without statistical significance. In our study, among all patient demographic factors, only male sex showed a significant association with having malaria among febrile travelers. The majority (85%) of malaria patients were adult males, which is consistent with the result of previous studies in Saudi Arabia^21,24–26^ and Europe^27^. The majority (75%) of our study population were young adults (44%) and middle-aged adults (31.3%). This finding can be attributed to the fact that the majority of recruited expatriate manpower are adult males from malaria endemic areas^21,24–26^. Culturally, adult males in Saudi Arabia spend more time outdoors and travel more frequently than females, and thus are more exposed to mosquito bites than children and females. Furthermore, women in Saudi Arabia, a predominantly Muslim country, wear clothes that cover their entire body, which serves to protect them from mosquito bites. This explanation is applicable for the few autochthonous study cases.

Hospitalized and ER febrile patients were predictive of malaria in our study. Similarly, it was reported that malaria was the major infection among febrile travelers hospitalized or presenting to ER^3,28^. This may be due to the fact that malaria is an acute condition, thus most febrile patients presented to the hospital by the Emergency Room. Consequently, the probability of malaria should be excluded for all cases presenting at ER who come from endemic areas and complained of acute fever.

All febrile traveler patients who presented at KFHU, either coming from malaria endemic areas or for whom malaria was clinically suspected, had their blood films microscopically screened for malaria with expert microscopists. Malaria rapid diagnostic test (RDT) does not require equipment and requires less expertise than malaria microscopy, however it is not cost effective in comparison to malaria microscopy as it misses low parasitemia and gives false positive cases due to cross reactivity^29,30^. Malaria RDT is more suitable for primary care centers with non-expertise microscopists and limited facilities. Microscopy for malaria diagnosis in tertiary hospitals, with expert microscopists, is a cost-effective method to determine the *Plasmodium* species, stages, and parasite quantification in positive malaria cases^29,30^.

To improve the prevention and elimination of malaria, it is important to understand the dynamics of malaria seasonality. In the current study, malaria cases were reported throughout the year, with annual seasonality and peaking in September, and most cases in early summer (June) and fall (August, September and October) without statistical significance. This may be attributed to the increase in travel in these months by Saudi nationals, and Indian, Pakistani and African expatriates, returning back to Saudi Arabia after spending summer vacation in their respective countries, when malaria is peaking and there is high anopheles mosquito biting and abundance^31,32^. Climate drivers, such as temperature, vegetation coverage and rainfall pattern, are greatly variable among different localities and influence malaria transmission, mosquito population ecology, parasite rate and inoculation rate; however, their role is not clear^31–34^. Thus, our data most probably reflects a seasonality of travelling rather seasonality of malaria transmission.

The current study could have benefited from more details concerning the study populations’ travel movements. Furthermore, as this is a single institute study, it is not representative of all cases of malaria in the Eastern Province of Saudi Arabia.

## Conclusion

There was very low malaria prevalence among the febrile patients who attended KFHU; all were travel-related malaria cases, except for one due to transfusion malaria. Most of the confirmed malaria cases at KFHU came from endemic countries, however a very low percentage of indigenous malaria cases existed as well. No patient data could predict malaria. Based on our findings, patients with acute fever coming from endemic areas or having received blood transfusion, should be screened for malaria. In the presence of a potential vector, travel-associated malaria in non-malaria endemic areas should be monitored to guide planning and implementation of control strategies.
